# 2023 MCBK global meeting—Lightning talk abstracts

**DOI:** 10.1002/lrh2.10443

**Published:** 2024-07-06

1. Afzal, Muhammad: Building Smart Biomedical Knowledge Repository.
2. Aljibe, Miguel: Lessons from Applying the WHO SMART Guidelines in the Philippines.
3. Bajra Bajracharya, Ravi: Semantic representation of Biomedical Knowledge as a Knowledge Graph.
4. Bhasin, Neha: Computable Biopsychosocial Knowledge for Psychiatric Healthcare.
5. Boisvert, Peter: “We are not what we know but what we are willing to learn”: Extensible metadata for CBK.
6. Carlyle, Ruth: Health and digital literacy to underpin CBK: NHS England data + experience.
7. Chen, Shuaipu: Research on Approaches of Computable Knowledge in Biomedical and Health Sciences Based on Metaknowledge and Knowledge Scenario.
8. Dehnbostel, Joanne: Ontology Management on FHIR: the Scientific Evidence Code System (SEVCO).
9. Flynn, Allen: Economic Incentives and Upholding the Rights of CBK Creators.
10. Hanauer, David: EMERSE: Enabling Access and Utilization of Unstructured EHR Notes for Computable Biomedical Knowledge in Healthcare Settings.
11. Hao, Jianguo: Hierarchical Contrastive Learning‐Enhanced Multi‐label Classification for Chest X ray Radiographs.
12. Jing, Xia: Developing computable clinical decision support rules from CDC vaccination recommendations—how many mediate layers are needed?
13. Kang, Inwon: Exploring the Use of Large Language Models for Generating Decision Logic and Smart Contracts for Healthcare Processes from Natural Text.
14. Khoury, Rami: Standards, Interoperability, Metadata, Technical Models & Infrastructures for CBK.
15. Kong, Guilan: Computable phenotyping for Type 2 diabetes.
16. Landis‐Lewis, Zach: Using vignettes for mobilization of computable knowledge.
17. Liu, Wentie: Machine learning‐based in‐hospital mortality prediction for ICU patients based on clinical data in the first 3 hours of sepsis.
18. Martinez, Joshua: PhacoTrainer: A Deep Learning Web App to Enhance Cataract Surgical Training.
19. Morris, Alan: Automation of Evidence‐based care creates a Learning Healthcare System.
20. Morrissette, Katelin: Implementation of Data Element Enhanced Clinical Documentation.
21. Osheroff, Jerome: A Pain/Opioid Use Case for Mobilizing CBK: Driving Learning Health Systems.
22. Scott, Philip: NICE computable implementation guidance – next steps.
23. Sen, Simmi: Evaluation of Computable Performance Metrics for Cataract Surgery Enabled by Artificial Intelligence.
24. Shi, Tongyue: ICU Mortality Prediction Based on Patient Subgroups Identified Using Multivariate Time‐Series Clustering.
25. Šuster, Simon: Using Machine Learning and Natural Language Processing to Structure Medical Evidence and Grade its Quality.
26. Tsafnat, Guy: Evidence Hub: a place to exchange knowledge and form communities.
27. Viguera Moreno, Minerva: Using REDCAP to support the development of a Learning Healthcare System for Multiple Sclerosis patient management.
28. Wood, Hannah: Prompting Large Language Models to construct Boolean search strategies for Google and advanced search databases.
29. Wyatt, Jeremy: Which computable biomedical knowledge objects will be regulated as a medical device?

## BUILDING SMART BIOMEDICAL KNOWLEDGE REPOSITORY

Muhammad Afzal, School of Computing and Digital Technology, Birmingham City University

muhammad.afzal@bcu.ac.uk

Contemporary scientific communication relies heavily on document‐based systems like journal articles, books, and reports for sharing research findings. However, large documents limit opportunities for efficient knowledge dissemination due to limitation in processing of different subsections within a document to understand the meaning of information units. This research aims to develop a smart repository that moves beyond documents and introduces smaller, computable units of knowledge. By assessing biomedical data sources, we will build a repository to make scientific knowledge more representable, computable, and shareable. The rationale is to enhance how researchers communicate and manage information in the rapidly evolving digital era.

The work focuses on developing a new repository that goes beyond the document‐based paradigm by fusing biomedical and health and life sciences data sources, such as PubMed Central. New protocols and methods will be designed to identify relevant sections in the documents to extract smaller knowledge units. The proposed repository with key features storage, retrieval, representation, and sharing will be optimized for the granular units. Integration strategies with existing platforms like PubMed will be devised. Usability testing will refine the interface to boost engagement. Interoperability mechanisms will ensure compatibility with existing systems.

By enabling scientific knowledge to be shared in smaller units, this repository has the potential to revolutionize scientific communication and collaboration. Breaking down information into granular components is expected to create new opportunities for innovation, discovery, and the development of advanced analytics tools. The repository will facilitate efficient access to health evidence, benefiting researchers, clinicians in creating systematic reviewers that require rapid evidence synthesis. Further, the computable units extracted from documents could be modeled into interoperable resources like FHIR, thereby support the Evidence Based Medicine on FHIR (EBMonFHIR) project is extending FHIR to provide a standard for machine‐interpretable exchange of scientific knowledge. This would also allow developers to build innovative AI systems for objectives such as diagnostic and treatment support.

By reducing the need for manual effort in finding and formatting evidence, the repository will pave the way for automating knowledge synthesis and management and will empower various stakeholders with enhanced efficiency, interoperability, and analytical capabilities to progress research and practice.

## LESSONS FROM APPLYING THE WHO SMART GUIDELINES IN THE PHILIPPINES

Miguel Aljibe, University of the Philippines

moaljibe@up.edu.ph

Alvin Marcelo, University of the Philippines‐Manila

admarcelo@up.edu.ph

Janus Ong, University of the Philippines‐Manila

jpong@up.edu.ph

Geohari Hamoy

geohari.hamoy@gmail.com

The World Health Organization released the SMART Guidelines to advise member countries on a systematic approach to converting narrative policies into machine‐readable formats. In the Philippines, a group of researchers attempted to apply the guidelines to the national viral hepatitis program. Several lessons were learned. First, government sponsorship is crucial at the outset as the effort of conversion can be substantial and confidence that the output will eventually be promulgated is essential. Second, consensus building is important to ensure that all stakeholders have been consulted and that the semantics are understood and accepted by all. Third, international standards such as HL7 FHIR and SNOMED play important roles. They must be made available for all to use. Lastly, constant communications among stakeholders (see consensus building in #2) will enable data exchange because of the trust built with open channels.

## SEMANTIC REPRESENTATION OF BIOMEDICAL KNOWLEDGE AS A KNOWLEDGE GRAPH

Ravi Bajra Bajracharya, CEO/Knowledge graph engineer, datum.md

ravi@datum.md

1. Biomedical knowledge challenge

Healthcare industry arguably has an abundance of biomedical knowledge bases for different domains and sub‐domains within. However, re‐usability of these knowledge sources is fairly limited to their immediate scope of use. One reason for this is that knowledge bases are not released in an interoperable and reusable format and often require lot of domain knowledge to be able to pre‐process and integrate these into applications. Hence there is a lot of value in following the FAIR (Findable, Accessible, Interoperable and Reusable) principles when publishing these knowledge sources. There is even more value in linking more than one disparate but related sources of biomedical knowledge such as clinical trials data set with related pub‐med resources for example. Moreover, still even more value is generated when we can link patient health data or study data to these biomedical knowledge sources particularly those containing RWE (Real World Evidence) knowledge base. But numerous challenges in the ecosystem including lack of adoption of interoperability standards contribute to the challenge of making biomedical knowledge bases accessible to a much broader application base in healthcare in general.

2 Knowledge Graph (KG) Solutions

Knowledge graphs are formal representation of knowledge bases as a network of nodes and edges where nodes represent biomedical concepts and edges link related nodes together to encode relationships between concepts in a knowledge base. A semantic knowledge graph treats each node as a resource with a unique URI and a semantic namespace and thereby normalizes concepts to similar semantic space across various sources of knowledge or information.

A knowledge graph can also be grounded on a set of primitives from which it is constructed. Knowledge graphs can also include a set of logical rules on top of which actual inferences can be based, for example, the human p53 gene is a subclass of the p53 protein class that is found in the organism human.

3 Biomedical KG construction and application

The primitives in our case can be health data standards, ontologies, terminologies, and vocabularies, which can be enriched using both structured and unstructured knowledge bases ranging from basic science, genomics data to clinical longitudinal records and population health demographics to community, environmental and behavioral research data.

The knowledge graph can infer new knowledge through logical reasoning or through applications of graph‐based algorithms or through analytics (descriptive or predictive), the result of which can further feed back into the knowledge graph there by creating a self‐learning feedback mechanism to grow the knowledge graph.

4 Summary

The use of semantic data representation in a knowledge graph platform has proven to expand the use cases of the represented knowledge base to broader applications in the areas of analytics and prediction promoting reuse and interoperability of underlying biomedical knowledge sources involved.

## COMPUTABLE BIOPSYCHOSOCIAL KNOWLEDGE FOR PSYCHIATRIC HEALTHCARE

Neha Bhasin, MD, MS, University of San Francisco

nbhasin@dons.usfca.edu

William J. Bosl, Digital Health Informatics Program, University of San Francisco, Center for AI and Medicine, University of San Francisco, Computational Health Informatics Program, Boston Children's Hospital, Harvard Medical School

wjbosl@usfca.edu

**Introduction**: Psychiatric disorders impose an enormous burden of disease on all populations of the world, a burden that is likely underestimated because of a failure to appreciate the interconnectedness of mental and physical health. While research in basic neuroscience has advanced considerably, translation of new knowledge into new treatments has been slow. Mental disorders generally emerge over time due to complex factors, creating opportunities for early intervention to redirect the developmental trajectories and to monitor the efficacy of treatments. Unfortunately, the data necessary to monitor neurodevelopmental trajectories relevant to psychiatric disorders are typically not collected in routine clinical care. Further, research to develop computational algorithms to model disease trajectories depends on these data that are not generally available from clinical sources. We propose creation of a new paradigm for a learning mental health system that is designed around a globally accessible personal psychiatric database that will support research in computational psychiatry and will evolve into clinical decision support systems.

**Objective**: The primary goal for this research is to create a new paradigm to collect mental health relevant data to develop algorithms for monitoring neurodevelopmental trajectories to enable early risk assessment and monitoring for mental disorders.

**Methods**: Our team and others are developing computational approaches that enable latent information from complex, multimodal data, including EEG as a functional brain measurement, to be extracted. Importantly, clinical validation of these computational methods will require standardized datasets from large, diverse populations. We are implementing an open and secure platform for brain specific data using open (FHIR) APIs that will enable this data to be shared with institutionally based EHRs. Our goal is to create a globally accessible cloud‐based personal mental health record for research in computational psychiatry that also seeks to create a brain‐health monitoring clinical paradigm. Consent for running algorithms on this data in a federated fashion will be mandatory. Our methodology integrates three components to create a computable mental health knowledge system: a mobile platform to collect EEG and other relevant data outside of the clinic, a FHIR‐based personal mental health database, and algorithms to compute neurodevelopmental risk trajectories from the database.

**Discussion**: Our previous research has recruited research cohorts to identify biomarkers for neurodevelopmental disorders in children from EEG recordings. To transition this work from the laboratory to practice, longitudinal data is needed for clinical validation studies. These data are not typically collected in routine pediatric checkups, necessitating a new paradigm for acquiring the relevant data. We are proposing a new kind of learning health system in which the need for ecologically valid research data will drive the creation of a new patient‐centric approach to mental health monitoring. Our initial focus will be on neurodevelopmental disorders in children.

**Conclusion**: Care for brain disorders may be advanced by patient‐centered longitudinal research that implements Computable Biopsychosocial Knowledge for psychiatry through FHIR‐based, brain‐specific personal health record systems, together with algorithms tested and developed on that database. An initial pilot implementation for childhood neurodevelopmental disorders is being implemented to demonstrate the system.

## “WE ARE NOT WHAT WE KNOW BUT WHAT WE ARE WILLING TO LEARN”: EXTENSIBLE METADATA FOR CBK

Peter Boisvert, Department of Learning Health Sciences, University of Michigan

pboisver@umich.edu

Marisa Conte, Department of Learning Health Sciences, University of Michigan

meese@umich.edu

Allen Flynn, Department of Learning Health Sciences, University of Michigan

ajflynn@umich.edu

Charles P Friedman, Department of Learning Health Sciences, University of Michigan—on behalf of the Knowledge Systems Lab

cpfried@umich.edu

Metadata is essential to achieve FAIR (Findable, Accessible, Interoperable and Reusable) computable biomedical knowledge (CBK). The Mobilizing Computable Biomedical Knowledge (MCBK) community has defined categories of metadata^[1]^ and supports ongoing efforts to develop a minimal metadata model for CBK. Complementing this work, we recognize an emerging role for extensible metadata, which can be generated by both human‐ and non‐human actors throughout the lifecycle of a CBK artifact.

Technical methods for enabling extensible metadata are well‐known, including Protégé for ontology creation and management, and the CEDAR workbench for authoring metadata. Existing domain and infrastructural metadata can capture various perspectives on CBK artifacts or collections, including domain‐specific (Human Phenotype Ontology) or provenance (PROV‐O) ontologies, technology‐specific metadata (Maven coordinates), and so on. In a linked‐data world, these should all be linkable and interoperable in the context of a particular instance or class of CBK, and its stakeholders.

While many aspects of CBK pose socio‐technical challenges, we see extensible metadata as falling largely on the socio‐ side. Extensible metadata can address emerging issues in CBK creation, implementation and management:

- CBK is developed by and for diverse users.

There are many types of CBK, developed for a variety of users, use cases, and communities of practice. CBK creators, implementers and adopters may have different needs and interests that may be reflected by different types of metadata. Extensible metadata allows users and communities to reflect the fitness for purpose of CBK to their own domains, uses and contexts.

- No single standard can describe all useful and relevant information:

While standards‐based metadata, including schemas and standard terminologies and ontologies are important, it's unlikely that any single standard will cover the diversity of needs and uses of various users and communities.

- CBK is dynamic—as it changes, so should its metadata:

Enabling the enrichment of CBK metadata over time reflects both the changing knowledge and the changing ways in which that knowledge is understood and used beyond its initial conception.

- Communities of practice may benefit from means to document the use, evaluation, and endorsement of CBK:

When knowledge is embedded in a network, others can use and react to the knowledge in a variety of ways. These interactions can become metadata, informing the use of CBK by others. This is an existing practice in scholarly communication, e.g., the tracking and reporting of citation patterns.

- Trust emerges from networks and communities:

Trust in CBK is essential for its use, dissemination, and reuse. Trust is also essentially a function of networks and community, not of a CBK artifact itself. Extensibility allows communities to enrich metadata with elements that are essential for trust by that community, increasing the potential for increased implementation and use.

Ongoing efforts to develop standards and models for CBK metadata should also include ways to promote extensible metadata. Extensibility acknowledges the plasticity of both CBK and its uses/users, and the enriched metadata provided by diverse communities or other agents may increase the adoption, usefulness, and reusability of CBK.

^1^ Alper BS, Flynn A, Bray BE, Conte ML, Eldredge C, Gold S, et al. Categorizing metadata to help mobilize computable biomedical knowledge. Learn Health Syst 2021;n/a(n/a):e10271.

## HEALTH AND DIGITAL LITERACY TO UNDERPIN CBK: NHS ENGLAND DATA + EXPERIENCE

Ruth Carlyle, NHS England

ruth.carlyle@hee.nhs.uk

Application and use of computable biomedical knowledge depends upon digital skills and health literacy, the ability to access, assess and use health information.

This lightning talk will share data from England on variations in health literacy and digital connectivity. At a national level, 43% adults aged 16–25 struggle to understand health information that uses words. When numbers are added, 61% adults struggle—as most health information in practice combines words and numbers, this means that the majority of the population struggle even when health information is in print (Rowlands et al 2015). If members of the public are to contribute to and benefit from the personalisation of health information made possible by computable biomedical knowledge, they need to understand the information and to trust both the sources and the usage.

The national NHS Knowledge and Library Services team (now part of NHS England) provides strategic leadership for NHS knowledge and library services in England. The aim in its strategy is that ‘NHS bodies, their staff, learners, patients and the public use the right knowledge and evidence, at the right time, in the right place, enabling high quality decision‐making, learning, research and innovation, to achieve excellent healthcare and health improvement’ (Knowledge for Healthcare, 2021). As part of delivering this aim, we have a workstream on health literacy and patient information, developing national partnerships and creating and spreading tools.

As a tool to understand health literacy, the team commissioned the University of Southampton to reanalyze literacy and numeracy data as geodata, to show local variation. This lightning talk will give a brief insight into both health literacy geodata and mapping of digital connectivity, with its implications for computable biomedical knowledge.

The talk will also introduce a national partnership with CILIP (the professional body for librarians and information professionals in the UK), Libraries Connected (overseeing public/community libraries) and Arts Council England. Through the partnership, community‐based public libraries and prison libraries have worked with members of the public to increase confidence in health literacy and provide skills and digital hubs to interact with health information in digital forms. Learnings from this activity demonstrate the role that libraries and library staff in health and community settings can provide in increasing the health literacy and digital literacy for the public on which use of computable biomedical knowledge depends.

## RESEARCH ON APPROACHES OF COMPUTABLE KNOWLEDGE IN BIOMEDICAL AND HEALTH SCIENCES BASED ON METAKNOWLEDGE AND KNOWLEDGE SCENARIO

Shuaipu Chen, School of Information Management, Wuhan University, Institute of Big Data, Wuhan University

neochenspu@whu.edu.cn

Yuxing Qian, School of Information Management, Wuhan University, Institute of Big Data, Wuhan University

qianyuxing@whu.edu.cn

Zhenghao Liu, School of Information Management, Wuhan University, Institute of Big Data, Wuhan University

zhenghaoliu@whu.edu.cn

In the application of the Data‐Information‐Knowledge‐Wisdom model in biomedical and health sciences, the computation of the value of knowledge becomes pivotal in empowering decision‐making as a tangible manifestation of wisdom.

We propose a specific method for knowledge computation, drawing from both metaknowledge theory and scenario theory. Metaknowledge theory highlights the value of knowledge not only in its content but also in its cognitive states. To address the complexity of knowledge representation, we first redesign the organizational form of knowledge content, as the traditional triplet format proves inadequate. Consequently, we introduce the concept of knowledge scenario based on scenario theory, intending to enrich knowledge with additional attributes beyond the traditional triplet format. These attributes are represented as scenario attributes that encompass both physical and information spaces, emerging through the process of knowledge generation and application. This step accentuates the computation of knowledge content applicability. Moving forward, we proceed to compute knowledge cognitive states by analyzing the certainty strength of the knowledge itself and the supporting strength of external evidence. Through the integration of these two aspects, we successfully achieve knowledge computation.

To verify the effectiveness of our proposed method, we conduct two experiments. Firstly, we apply the method to biomedical and health sciences, specifically focusing on Mild Cognitive Impairment (MCI). We construct a scenario‐based knowledge graph and establish rules to explore the multi‐angle knowledge association characteristics of fusibility, inheritance, and inference. By retrieving knowledge based on scenario matching, we significantly enhance the applicability of the knowledge. Secondly, we employ ChatGPT, a representative large language model, and integrate our method as a prompting engineering following a specific chain of thought. By applying this approach to the diagnosis of MCI, we effectively mitigate the occurrence of hallucinations in the large language model, substantially improving the reliability of knowledge.

## ONTOLOGY MANAGEMENT ON FHIR: THE SCIENTIFIC EVIDENCE CODE SYSTEM (SEVCO)

Joanne Dehnbostel, MS, MPH, Computable Publishing LLC, Scientific Knowledge Accelerator Foundation

jdehnbostel@computablepublishing.com

Brian S. Alper, MD, MSPH, Computable Publishing LLC, Scientific Knowledge Accelerator Foundation

balper@computablepublishing.com

Khalid Shahin, BA, Computable Publishing LLC, Scientific Knowledge Accelerator Foundation

kshahin@computablepublishing.com

Joshua Richardson, PhD, MS, MLIS, FAMIA, RTI International

j.e.richardson@outlook.com

Standard terminologies facilitate unambiguous communication in many domains. However, there are no globally accepted and adopted standard terminologies for reporting scientific knowledge. A global effort started in 2020 to define standard terminologies (in the form of code systems) for four scientific knowledge concepts: Study Design, Risk of Bias, Statistic Type, and Statistical Model.

The effort created a Code System Development Protocol to support global development of terminologies for exchanging scientific evidence. Initial steps of the protocol included (1) assembling expert working groups with people from more than 25 countries, (2) identifying 23 commonly used tools and systems for which standard terminology would be useful, (3) drafting 368 non‐redundant concepts to become display terms for the four code systems, (4) identifying 27 ontologies with related terms and definitions, and (5) mapping available terms and definitions for the draft concepts. (Alper BS, Dehnbostel J, Afzal M, Subbian V, Soares A, Kunnamo I, Shahin K, McClure RC, For the COVID‐19 Knowledge Accelerator (COKA) Initiative. Making Science Computable: Developing code systems for statistics, study design, and risk of bias. Journal of Biomedical Informatics 2021 Mar;115:103685. https://doi.org/10.1016/j.jbi.2021.103685).

We consolidated these efforts into a single Scientific Evidence Code System (SEVCO), developed tooling to facilitate the work (including FEvIR®: CodeSystem Builder/Viewer and FEvIR®: My Ballot), and streamlined the protocol to enable more efficient development processes (Alper BS, Dehnbostel J, Lehmann H, Whaley P, Wilkins KJ, Tufte J, Yurk RA, Ojha N, Afzal M. For the COVID‐19 Knowledge Accelerator (COKA) Initiative. Scientific Evidence Code System Development Protocol. Created November 16, 2021. Last revised December 8, 2021. Available at: https://tinyurl.com/SEVCOprotocol).

The SEVCO development is available for open viewing at. https://fevir.net/resources/CodeSystem/27270#TOP and anyone can comment on any term. Participation in terminology deliberations is also open to anyone. You can self‐select to join the SEVCO Expert Working Group at https://fevir.net/resources/Project/27845 and as terms are open for voting, you can vote Yes or No and/or provide comments for what changes may be needed. Terms are passed when they receive 100% agreement with at least five votes, and negative votes lead to discussion and modification until they reach approval.

As of July 18, 2023, SEVCO has 595 terms, of which 342 (57.5%) have unanimous approval.

Once completed, the code system should more effectively facilitate identifying, processing, and reporting research results and the reliability of those results. More efficient and detailed scientific communication will reduce cost and burden and improve health outcomes, quality of life, and patient, caregiver, and healthcare professional satisfaction.

SEVCO is developed by the Health Evidence Knowledge Accelerator (HEvKA), which is an open, virtual group to accelerate identifying, processing, and disseminating computable biomedical knowledge, especially related to clinical research. HEvKA efforts inform and develop standards, terminologies, and tools for computable expression of evidence and guidance. You can participate through any of 15 open virtual weekly meetings. Details at https://tinyurl.com/HEvKA

## ECONOMIC INCENTIVES AND UPHOLDING THE RIGHTS OF CBK CREATORS

Allen Flynn, University of Michigan Medical School and School of Information

ajflynn@umich.edu

What rights should CBK creators and Metadata Contributors enjoy, and how will those rights be upheld? With written knowledge production, journals identify and authenticate authors, uphold standards via peer review, and maintain rights. Namely, via journals, rights of attribution and integrity of authors' works are upheld, incentivizing publication.

To have a trusted and economically viable online CBK ecosystem, identification, authentication, and attribution (IAA) of all creators and contributors is required. Yet the online world that has evolved to date fosters anonymity, not IAA. In his 2016 piece “How to fix the internet”^1^, Walter Isaacson prescribed changes to counter anonymous malicious online actors. We apply Isaacson's ideas to envision a trusted online CBK ecosystem that incentivizes voluntary, verifiable IAA.

Voluntary, verifiable IAA enables creators of CBK artifacts and metadata to benefit when others use what they produce. By upholding creators' rights of attribution and the integrity of CBK works, creators can benefit via recognition and payments. In addition, for economic viability to be achieved, these creators must be shielded from liability that cancels out such benefits. Only when creators can gain from participating in a future CBK ecosystem should we expect routine compliance with voluntary, verifiable IAA to follow.

Therefore, to be trusted and economically viable, a CBK ecosystem must:

1. Establish corresponding CBK and metadata production lifecycles.
2. Incorporate legally enforceable digital signatures by individual creators into CBK and CBK metadata production lifecycles to enable IAA.
3. Enable creators to be recognized and given authoring credit for their works.
4. Enable micropayments from users to creators.
5. Insure creators against losses when others use their CBK creations.

We imagine these five items are necessary, if not sufficient, to establish suitable economic incentives for any viable future CBK ecosystem.

^1^ Isaacson W. How to fix the internet. The Atlantic. 2016 Dec;15.

## EMERSE: ENABLING ACCESS AND UTILIZATION OF UNSTRUCTURED EHR NOTES FOR COMPUTABLE BIOMEDICAL KNOWLEDGE IN HEALTHCARE SETTINGS

David Hanauer, University of Michigan

hanauer@umich.edu

Lisa Ferguson, University of Michigan

ferglisa@med.umich.edu

Kellen McClain, University of Michigan

mcclaink@med.umich.edu

Guan Wang, University of Michigan

wanggu@med.umich.edu

Approaches for achieving computable biomedical knowledge (CBK) in the clinical domain often require the use of unstructured (free‐text) clinical notes from electronic health records (EHRs). There remain significant challenges for broad use of unstructured clinical data, in part because many natural language processing (NLP) and text retrieval/processing systems are too complex to use for non‐technical users. Our team has been developing tools for enabling non‐technical research and operational teams within healthcare settings to securely access and utilize unstructured EHR notes for a variety of purposes ranging from clinical research to quality improvement in healthcare systems.

EMERSE—the electronic medical record search engine—is a text retrieval and text processing system to help “democratize” the use of free text data in EHR notes, with a special emphasis on usability for non‐technical users. EMERSE has many features specifically designed to support biomedical research, including robust query expansion that can leverage similar terms from multiple vocabularies and ontologies (e.g., Human Phenotype Ontology, Orphanet Rare Diseases, the Gender, Sex, and Sexual Orientation Ontology, and more). EMERSE securely enables network‐based searches to obtain obfuscated patient counts across participating institutions. EMERSE also supports collaboration and re used of search terms through a feature in which collections of terms/phrases can be shared and re‐used by other teams. EMERSE also groups notes by patients, making it easier to identify cohorts for tasks such as eligibility determination for clinical trials.

New development work underway includes the additional of named entity recognition (NER) and coding notes to the Unified Medical Language System (UMLS) using Concept Unique Identifiers (CUIs). Such an approach will allow users to search for terms and concepts interchangeably. The ability to consider negation status, uncertainty, and whether the text is about the patient or another individual is also planned. EMERSE has been in constant development since 2005 and is now operational at multiple large academic medical centers across the United States and Europe. It is available at no‐cost under and open source license.

## HIERARCHICAL CONTRASTIVE LEARNING‐ENHANCED MULTI‐LABEL CLASSIFICATION FOR CHEST X‐RAY RADIOGRAPHS

Jianguo Hao, National Institute of Health Data Science, Peking University, Institute of Medical Technology, Health Science Center of Peking University

jianguo.hao@bjmu.edu.cn

Shichao Fang, King's College London

shichao.fang@kcl.ac.uk

Qing Li, Advanced Institute of Information Technology, Peking University

blacknepia@dingtalk.com

Guilan Kong, National Institute of Health Data Science, Peking University, Institute of Medical Technology, Health Science Center of Peking University, Advanced Institute of Information Technology, Peking University

guilan.kong@hsc.pku.edu.cn

Radiological examination is an effective and valuable method for thoroughly inspecting a patient's chest. Clinical data, which can provide valuable supervisory information for machine learning algorithms, is commonly stored in electronic health records (EHRs). Chest radiographs often contain multiple pathologies, making the diagnosis of these multi‐label medical images without precise annotations particularly challenging and time‐consuming. This study endeavored to combine a hierarchical clustering method called formal concept analysis (FCA) and a contrastive learning paradigm to learn enhanced discriminative representations from EHR data and chest x‐ray radiographs (CXRs), with the aim of offering support for downstream tasks, such as similar image retrievals.

The data source utilized in this study is the Medical Information Mart for intensive care chest x‐ray (MIMIC‐CXR) dataset, known as one of the largest de‐identified publicly accessible repositories of CXRs for clinical data analysis. Its de‐identified structured EHR data contains demographic information, concise clinical interpretations, and meta‐data such as orientations of CXRs. It should be noted that each patient may undergo multiple radiological examinations, with each preliminary diagnosis being documented in a free‐text radiology report.

To encode CXRs, we leveraged a deep learning model, EfficientNet, as the backbone network. The model was initialized with pretrained weights obtained from the ImageNet dataset and then fine‐tuned for transfer learning using contrastive learning to adapt the MIMIC‐CXR dataset. The model further incorporated a pretext task employing a hierarchical clustering algorithm to cluster CXRs. Categorical EHR data extracted from free‐text reports, such as pathologies or CXR findings were served as a source of supervisory information. The MIMIC‐CXR dataset was split into training, test, and validation subsets. Subsequently, FCA approach generated a hierarchical clustering graph that clustered the CXRs based on various sets of supervisory information. In the generated graph, clusters were systematically organized and interconnected based on their mutual correlations of supervisory information. It could facilitate a coherent representation of relationships and dependencies among the clustered CXRs, providing valuable insights into their similarities and dissimilarities. Given an anchor CXR, the graph classified indexed CXRs as either positive or negative cases by considering the relationship between their supervisory information. To develop the instance discrimination model using contrastive learning, triples consisting of anchor CXRs together with their corresponding positive and negative CXRs, were formed in the training process. These encoded triples were designed to continuously optimize the model by a contrastive loss to get similar CXRs closer and pull away dissimilar ones. This optimization process made the learned representation space of CXRs more discriminative. Compared to self‐supervised contrastive learning, the proposed hierarchical contrastive learning performed better in the downstream task of cross‐modal retrieval for similar cases. The average retrieving precision of the top 5 retrieved similar cases increased from 76.80% to 81.01%.

To summarize, we proposed a supervised hierarchical contrastive learning approach to learn discriminative representations from multi‐label CXRs. By combining contrastive learning with hierarchical clustering, the proposed model reinforces the hierarchical supervision and makes all the studied cases well represented, and thus enables better discrimination of multi‐label CXRs.”

## DEVELOPING COMPUTABLE CLINICAL DECISION SUPPORT RULES FROM CDC VACCINATION RECOMMENDATIONS—HOW MANY MEDIATE LAYERS ARE NEEDED?

Xia Jing, Clemson University

xjing@clemson.edu

Richard D. Boyce, University of Pittsburgh

rdb20@pitt.edu

Hua Min, George Mason University

hmin3@gmu.edu

Yang Gong, University of Texas Health Science Center at Houston

yang.gong@uth.tmc.edu

James J. Cimino, the University of Alabama at Birmingham

jamescimino@uabmc.edu

Dean F. Sittig, University of Texas Health Sciences Center at Houston

dean.f.sittig@uth.tmc.edu

Clinical decision support systems (CDSS) have played a critical role in delivering safe, efficient, and quality clinical care. Rule‐based CDSS have a long history of use in large medical institutions because they need to maintain ever‐evolving rules. However, sharing computable CDSS rules across institutions has yet become commonplace. Our international collaboration group aims to use ontology to develop computable CDSS rules, particularly for resource‐limited primary care settings. The work could provide usable, maintainable, up‐to‐date, and computable CDSS rules for primary care settings that lack in‐house IT professional support. We have used CDC‐recommended vaccination schedules (≤18 years, 2022 version, five tables with annotations) as the starting CDSS rules.

Translating the vaccination recommendations (start point) directly into machine‐processable formats (endpoint) is unrealistic. Several mediate layers are needed between the start and endpoints; for example, a detailed tabular format compiled from the current CDC vaccination recommendation schedules is required; then, a detailed and thorough version represented in formal language formats can be easily converted to different programming languages. We aim to use unambiguous formats to represent the human‐readable CDSS rules from tabular formats to one that programmers can use to develop machine‐processable formats accurately. This strategy also minimizes errors in generating the CDSS rules in different programming languages.

We have completed the first mediate layer with over 465 rules for 19 vaccines in tabular format. We are currently generating the second mediate layer: a detailed and accurate version of the CDSS rules in formal languages, such as Web ontology language (OWL), clinical quality language (CQL), or Drools. The formal language formats can be translated into programming languages based on the deployment system requirements. Meanwhile, we are constructing a CDSS ontology to organize the mapping between concepts and individual terms across terminologies, both of which are used in representing the CDSS rules in mediate and final versions. OWL can provide reasoning potential; however, there are limitations to representing the complex and exact logical expressions typical of CDSS rules. CQL seems more promising in representing logic expressions. However, downstream use needs further demonstrations. Drools has been used in generating business logic and rules; however, like OWL and CQL, there is a deep learning curve to overcome in using Drools and setting up the work environment.

Although there will be challenges along the way, this is a feasible pathway for generating computable CDSS rules, a critical milestone in achieving true interoperability. Converting the 5‐table CDC vaccination recommendations into detailed tabular formats and then to CDSS rules in formal languages bridges human comprehensible representations and machine‐processable formats with explicit, clear, accurate representations of CDSS rules. It is a step toward achieving truly interoperable patient records, a long‐time dream yet to be realized in healthcare IT.

## EXPLORING THE USE OF LARGE LANGUAGE MODELS FOR GENERATING DECISION LOGIC AND SMART CONTRACTS FOR HEALTHCARE PROCESSES FROM NATURAL TEXT

Inwon Kang, Rensselaer Polytechnic Institute

kangi@rpi.edu

William van Woensel, University of Ottawa

william.vanwoensel@gmail.com

Oshani Seneviratne, Rensselaer Polytechnic Institute

senevo@rpi.edu

We explore using Large Language Models (LLMs) to generate computable knowledge in the form of decision logic (Notation3, Clinical Quality Language) and smart contracts (Solidity) that encode health insurance processes found in natural text. We present an initial methodology that generates output at increasing levels of complexity and technical detail: i.e., with generation tasks (1) structured summaries, (2) formalized decision logic, and (3) smart contract code. The LLM's output at each level can support a domain expert or Web3 developer in authoring decision logic or smart contracts. We engineered our LLM prompts following best practice guides provided by OpenAI and Deep learning AI. We propose experiment metrics, including completeness, soundness, clarity, and syntax, to evaluate the LLM's output. Our evaluation employs three textual scenarios on health insurance processes, progressively increasing in difficulty in length and comprehensibility for non‐domain experts, sourced from Medicare's official booklet. Our findings indicate that the LLM performs well in generating structured textual summaries across all scenarios. However, for tasks (2) and (3) that generate decision logic and runnable smart contracts, implementing particular health insurance processes, we found that the model's output requires human oversight to ensure validity. Notably, the model does not apply basic conceptual modeling principles, meaning that when runnable, the generated code will not be sound (i.e., yielding false positives or negatives). Moreover, the most advanced scenario seems too complex for the LLM to generate a correct set of decision logic and code, with more complex conditions being outputted as natural language comments instead of code, and other conditions simply missing. However, one of our decision logic targets, Clinical Quality Language, has very poor syntax for all scenarios, likely due to the lack of online training data. Nevertheless, our experiments demonstrate the promise of LLMs in supporting the translation of process descriptions from dense natural text into formal decision logic and smart contracts, which may aid streamlining many complex processes.

## STANDARDS, INTEROPERABILITY, METADATA, TECHNICAL MODELS AND INFRASTRUCTURES FOR CBK

Rami Khoury, MD, American College of Emergency Physicians Board Member, Independent Emergency Physicians‐PC

rkhoury@iep-pc.com

Pawan Goyal, MD, MHA, American College of Emergency Physicians

pgoyal@acep.org

Dhruv Sharma, BA, MS, American College of Emergency Physicians

dsharma@acep.org

Data is driving the future of medicine. We've already seen the critical importance of real‐time insights to new and emerging health threats during the COVID‐19 pandemic, as well as the impact of health care trends and patterns of resource utilization. With the new Emergency Medicine Data Institute (EMDI), the American College of Emergency Physicians (ACEP) is rapidly moving emergency medicine to the forefront of data‐driven quality and practice innovation. This new initiative is poised to become a source of truth for all emergency medicine data. Harnessing the power of information that physicians are already recording, ACEP collates vital metrics from emergency departments nationwide to support research and grants, while enhancing value for emergency physicians, patients, and the broader health care community. The presenters will enumerate the current, state‐of‐the‐art, and future challenges facing emergency medicine that will be addressed by the EMDI, illuminated with examples of data use from other medical specialties and early success stories. Attendees will be given a sense of emerging trends in clinical, quality, and economic aspects of emergency care that demonstrate the value and power the EMDI brings to the specialty.

## COMPUTABLE PHENOTYPING FOR TYPE 2 DIABETES

Guilan Kong, National Institute of Health Data Science at Peking University, Advanced Institute of Information Technology, Peking Universit.

guilan.kong@hsc.pku.edu.cn

Jiayu Wang, Institute of Medical Technology, Peking University Health Science Center

jiayu_wang@stu.pku.edu.cn

Bixia Gao, Renal Division, Department of Medicine, Peking University First Hospital

sherrygao1021@126.com

Jinwei Wang, Renal Division, Department of Medicine, Peking University First Hospital

gslzwjw@163.com

Luxia Zhang, National Institute of Health Data Science, Peking University

zhanglx@bjmu.edu.cn

Minchao Liu, Department of Computer Application and Management, Chinese PLA General Hospital

13810756690@139.com

Diabetes is a serious public health problem around the world. Nowadays, the prevalence of diabetes in low‐ and middle‐ income countries (LMIC) has been rising, and the increase of disease burden of diabetes in LMIC is faster than high income countries. Moreover, various types of complications may come with diabetes, and thus a heavy economic burden has been put on both patient families and the society. Conventionally, diabetes was classified into two types—Type 1 and Type 2—which are also known as “Insulin Dependent” and “Noninsulin Dependent” diabetes, respectively. The treatment methods for Type 1 and Type 2 diabetes are different accordingly. In practice, due to the high degree of heterogeneity among diabetes, the clinical characteristics and outcomes of Type 2 diabetes patients are very different as well.

With the advances of big data, the concept of computable phenotypes was proposed to classify patients into different subtypes based on clinical data characteristics. The patients belonging to the same disease subtype may have similar complication risk, and the treatment strategies of patients with common clinical characteristics may be similar.

In diabetes, a data‐driven subgroup analysis was conducted on a Swiss diabetes cohort in 2018, and five subtypes were identified for diabetes patients based on six clinical variables, including glutamate decarboxylase antibodies, age at diagnosis, body mass index (BMI), HbA1c, and homoeostatic model assessment 2 estimates of β‐cell function (HOMA2‐B) and insulin resistance (HOMA2‐IR). Each subtype has different patterns in complication occurrence. Several studies have been conducted among different ethnicities around the world to validate the existence of the identified 5 subtypes of diabetes.

In this study, the clinical data of diabetes patients going to the clinic of or being admitted to a Grade A Class 3 hospital in Beijing from 2000 to 2020 was used as data source. As glutamate decarboxylase antibodies is a clinical variable related to Type 1 diabetes and rarely recorded in the electronic medical records (EMR) system, diabetes patients with the data of five clinical variables (age, BMI, HbA1c, HOMA2‐B and HOMA2‐IR) recorded around the time of diabetes diagnosis were included for analysis. According to the central point value of each clinical variable in the five subtypes identified by the Swiss study, the distance between the clinical vector (age, BMI, HbA1c, HOMA2‐B and HOMA2‐IR) of each patient and the central point vector of each subtype was computed, and then each patient was classified into the subtype with the shortest distance. Therefore, each patient included for analysis was assigned a subtype based on his or her clinical characteristics at diabetes diagnosis. Finally, the patterns of diabetic complication occurrence were analyzed for the studied diabetes patients, and similar disease progression patterns have been found for patients labeled with the same subtype.

The computable phenotyping tool developed in this study had the capability to aid physicians to grouping Type 2 diabetes patients, and thus has the potential to facilitate precision and personalized medicine. Ideally, a recommendation of clinical treatment methods can be provided for each diabetes subtype after further analysis.

## USING VIGNETTES FOR MOBILIZATION OF COMPUTABLE KNOWLEDGE

Zach Landis‐Lewis, University of Michigan

zachll@umich.edu

Allen Flynn, University of Michigan

ajflynn@umich.edu

Peter Boisvert, University of Michigan

pboisvert@umich.edu

Hana Chung, University of Michigan

hanach@umich.edu

Patrick Galante, University of Michigan

pamaga@umich.edu

Ayshwarya Jagadeesan, University of Michigan

ayshjag@umich.edu

Mobilizing CBK requires that its implementers and key stakeholders efficiently develop an understanding of its purpose and function. However, supporting learning about CBK can be challenging due to the complex and abstract nature of algorithms, knowledge graphs, and computational models. Furthermore, when diverse models are implemented in software applications, the effort required to learn about a model collection can become prohibitively high. Methods to better support learning about CBK for its successful mobilization are needed.

Vignettes are tutorial‐like technical documentation, written for learning about R statistical software packages. Vignettes make complex software easier to understand by providing examples of data and analyses with supporting discussion and interpretation. To our knowledge, a practice like that of developing vignettes for CBK has not been widely adopted, yet writing vignettes may benefit CBK developers, implementers, and stakeholders in promoting more efficient learning about models and model collections.

We have developed 10 vignettes for a model collection in a precision feedback system. The system, which has been implemented for anesthesia providers, prioritizes motivational feedback messages about care quality based on the preferences of feedback recipients. The model collection includes logic models, ontologies, and algorithms which interact through modules of a software pipeline, implemented in a web service to support clinical quality dashboard and email content.

Each vignette is implemented as a readme file in a public GitHub repository. We developed each vignette with two characters (i.e., personas) whose differing preferences result in the selection of different messages by the precision feedback system. The vignettes describe system components and models, and include links to external resources, such as ontology terms and classes in BioPortal.

Through the vignette development process, we have begun to recognize multiple types of value. Our driving purpose has been to provide an accessible resource for learning about the precision feedback system by system implementers and stakeholders. However, the process of writing the vignettes has resulted in enhanced team communication and improved organization of system development tasks.

Furthermore, we have begun using the vignettes to guide system testing activities, including optimizing system performance. As a result of these insights, we plan to develop the vignettes as documentation that is packaged with the model collection and for future software releases of the precision feedback system.

## MACHINE LEARNING‐BASED IN‐HOSPITAL MORTALITY PREDICTION FOR ICU PATIENTS BASED ON CLINICAL DATA IN THE FIRST 3 HOURS OF SEPSIS

Wentie Liu, National Institute of Health Data Science, Peking University

2211210777@stu.pku.edu.cn

Tongyue Shi, National Institute of Health Data Science, Peking University

tyshi@stu.pku.edu.cn

Shuai Jin, Department of Adult Care, School of Nursing, Capital Medical University

jinshuai@ccmu.edu.cn

Jianguo Hao, National Institute of Health Data Science, Peking University, Institute of Medical Technology, Health Science Center of Peking University

jianguo.hao@bjmu.edu.cn

Huiying Zhao, Peking University People's Hospital

zhaohuiying109@sina.com

Guilan Kong, National Institute of Health Data Science at Peking University, Advanced Institute of Information Technology, Peking University

guilan.kong@hsc.pku.edu.cn

Sepsis is life‐threatening organ dysfunction caused by a dysregulated host response to infection. It leads to high in‐hospital mortality, particularly for intensive care unit (ICU) patients. Identifying septic patients who are at high risk of in‐hospital mortality early on can aid ICU physicians in making optimal clinical decisions. This study aimed to develop machine learning‐based tools that can predict hospital mortality risk for septic patients in the ICU based on clinical data gathered in the first 3 hr of sepsis.

Fluid therapy represents a clinical treatment approach which focuses on maintaining fluid homeostasis through the supplementation or restriction of specific fluids. As a life‐threatening condition, early resuscitation volume is important for sepsis patients, and it impacts patient prognosis and outcomes. Regrettably, most existing predictive models for sepsis mortality have not incorporated the early resuscitation volume for analysis. In clinical practice, early fluid resuscitation is advocated for sepsis patients. The 2016 Surviving Sepsis Campaign guideline recommends that at least 30 mL/kg of intravenous (IV) crystalloid fluid should be given within the first 3 h of resuscitation to patients with sepsis. As can be seen from the guideline, the initial 3 h following the diagnosis of sepsis are considered as the critical “golden time” for early resuscitation. Therefore, in this study, the intervention of early resuscitation was incorporated in mortality risk prediction modeling. A comprehensive set of clinical variables, which were collected in the first 3 h of sepsis diagnosis, together with the volume of crystalloid fluid administered during this initial 3 h were included for analysis.

The Medical Information Mart for Intensive Care‐IV(MIMIC‐IV) database containing records of over 40 000 ICU patients admitted to the ICUs at Beth Israel Deaconess Medical Center between 2008 and 2019, was used as data source. The sepsis patient data extracted from MIMIC‐IV formed a large study population and the clinical information contained including demographics, lab tests, clinical assessments and medical treatments. Around 80% of sepsis data was used for model development and the remaining for model test.

In terms of analytics, several machine learning methods with good explainability, which have also demonstrated satisfied predictive capabilities in medicine, including Random Forest (RF) and eXtreme Gradient Boosting (XGBoost), together with multivariate logistic regression were used in this study. Finally, prediction performance of two machine learning‐based models was compared with that of the traditional logistic regression, and the prediction model with best performance was selected for clinical recommendation.

The predictive tool developed in this study will help to identify sepsis patients with high in‐hospital mortality risk at an early stage. Hopefully, it will aid ICU physicians to provide timely and optimal interventions, and thus may help to improve ICU patient outcomes and reduce in‐hospital mortality.

## PhacoTrainer: A DEEP LEARNING WEB APP TO ENHANCE CATARACT SURGICAL TRAINING

Joshua Martinez, Stanford University

joshuakm@stanford.edu

Cataracts are the leading cause of vision impairment globally, and cataract surgery is the most commonly performed surgery in the United States. Continuously enhancing surgical training is key for ensuring high surgical standards and maintaining excellent visual outcomes of surgery. Traditionally, surgical training and feedback is based almost solely on preceptor‐led real‐time feedback in the operating room. The advent of machine learning, specifically deep learning models, has the potential to augment surgical feedback by enabling more granular and highly objective analysis of surgical performance through analysis of routinely captured surgical video. In previous work, we developed deep learning models that could identify key surgical landmarks and the surgical steps being performed in cataract surgery videos, thereby offering a novel means of quantitatively assessing surgical skill. We present here an implementation of these models into an application called PhacoTrainer, which is a platform designed to provide cataract surgeons with objective, automated feedback on their surgical techniques, and to facilitate future research on cataract surgery.

The PhacoTrainer platform is a web‐based application to which users can upload cataract surgical video and receive insights into their cataract surgical performance. The platform deploys a deep learning model, a hybrid Convolutional Neural Network and Recurrent Neural Network, to uploaded videos to detect which surgeries include special surgical techniques or complications. The model outputs also enable calculation of the time spent on each step of surgery, which then is displayed in a dashboard visualizing the change in surgical times for each step as a surgeon accumulates more experience. The timeline of each surgical video is also automatically annotated with a frame‐by‐frame determination of which surgical step is being performed, to better allow surgeons to browse through surgical video. Thus, the feedback provided by PhacoTrainer based on these models equips surgeons with insightful metrics to monitor their surgical performance across multiple dimensions, identifying areas for potential improvement.

The PhacoTrainer platform heralds a significant advancement in cataract surgery training and transforms unstructured cataract surgical video into computable insights. By mobilizing deep learning to objectively analyze surgical videos, it provides surgeons with a tool to self‐evaluate their skill, track improvements, record surgical metadata, and ultimately enhance surgical outcomes. PhacoTrainer also makes high quality feedback available to all trainees, irrespective of geographical or institutional constraints. With its capability to accumulate a vast repository of metadata on cataract surgery, PhacoTrainer also promises to catalyze future research on cataract surgery, facilitating a more nuanced understanding of surgical techniques over time and cataract surgical training.”

## AUTOMATION OF EVIDENCE‐BASED CARE CREATES A LEARNING HEALTHCARE SYSTEM

Alan H. Morris, MD, University of Utah

alan.morris@hsc.utah.edu

For the replicable decision‐support group.

Clinical decision‐making is based on knowledge, expertise, and authority, with clinicians approving almost every intervention according to the Hippocratic model of clinical decision‐making. This is the starting point for delivery of “All the right care, but only the right care,” a quality goal yet unachieved because unaided clinicians suffer from human cognitive limitations and biases when decisions are based only on their training, expertise, and experience. Robust decision‐support tools that reduce unwarranted variation of clinician decisions and actions can improve healthcare. Current electronic health records (EHRs) are focused on results review, documentation, and accounting. EHRs are awkward, time consuming, and contribute to clinician stress and burnout. Decision‐support tools can reduce clinician burden and enable replicable clinician decisions and actions that personalize patient care. However, most current clinical decision support tools/aids lack detail and neither reduce clinician burden nor enable replicable clinician actions. Clinicians must provide subjective interpretation and missing logic, thus introducing personal biases and mindless, unwarranted, variation from evidence‐based practice. Replicability occurs when different clinicians, with the same patient information and context, come to the same decision and action. A subset of therapeutic decision support tools based on credible clinical outcome evidence exists as computer protocols (eActions), including closed‐loop systems, that lead to replicable clinician actions. eActions enable different clinicians to make consistent decisions and actions when faced with the same patient input data and context. In advanced modern healthcare delivery environments, eActions have overcome cognitive limitations of overburdened clinicians. eActions embrace good everyday decision‐making informed by evidence, experience, EHR data, and individual patient status. eActions can reduce unwarranted clinician variation, increase quality of clinical care and research, reduce EHR noise, and could enable a learning healthcare system.

Delivering best evidence‐care remains a vexing problem that is achieved only ~50% of the time in advanced healthcare systems. Evidence‐based guidelines can address only a small fraction of the types of clinical care. Underserved areas rarely can access state‐of‐the‐art evidence‐based guidelines in real time, and often cannot implement advanced guidelines. Care providers in such settings frequently do not have sufficient training or time to implement advanced guidelines. Widespread use of eActions will require surmounting current healthcare technical and cultural barriers and installing clinical evidence/data curation systems that could yield new or modified evidence‐based guidelines derived from comparative effectiveness clinical research carried out during routine healthcare delivery within true learning healthcare systems. Developing countries could likely benefit as well from focused applications using limited healthcare electronic systems and smart phones.

## IMPLEMENTATION OF DATA ELEMENT ENHANCED CLINICAL DOCUMENTATION

Katelin Morrissette, University of Vermont Medical Center

katelin.morrissette@uvmhealth.org

Many important components of medical decision making, such as diagnostic certainty, interventions considered but avoided, or patient input in the management decisions are challenging to measure from existing data elements in the medical record. We present a method to build custom data elements to reflect these components of medical management and describe the implementation process. New innovations in medical management may not be represented in traditional elements of the electronic health record and will rely on these customized data elements as well. For example, in critical care medicine the phase of care before a patient is admitted to an intensive care unit (ICU) can be thought of as peri‐ICU. This peri‐ICU phase is when interventions which may avert an ICU admission, or define initial diagnosis and management occur. We have used features of the Epic EHR to create computable knowledge customized to this specific phase of care and a critical care consult service. The consult service bridges hospital departments, utilizes tele‐health, and seeks to treat critical illness outside the walls of an ICU. It was important to understand the impact on resource utilization as well as medical decision making and required customized data to be easily reported, shared, and improved over time. We use this case example to demonstrate feasibility and the implementation process for how computable data elements can be customized for specific clinical scenarios, rapidly deployed in line with clinical needs, and has potential to stimulate data sharing for concepts that were previously ambiguous and difficult to quantify.

## A PAIN/OPIOID USE CASE FOR MOBILIZING CBK: DRIVING LEARNING HEALTH SYSTEMS

Jerome A. Osheroff, TMIT Consulting, LLC and University of Utah Department of Biomedical Informatics and Department of Veterans Affairs

josheroff@tmitconsulting.com

Brian S. Alper, Computable Publishing LLC

balper@computablepublishing.com

Philip Barrison, University of Michigan Medical School Department of Learning Health Sciences

phbarris@med.umich.edu

Mary Butler, University of Minnesota

butl0092@umn.edu

Joshua E. Richardson, RTI International

j.e.richardson@outlook.com

Ken Rubin, University of Utah Department of Biomedical Informatics; Department of Veterans Affairs

ken.rubin@utah.edu

Janice Tufte, Hasannah Consulting

janicetufte@yahoo.com

for the Pain/Opioid LHS Learning Community https://docs.google.com/document/d/1J_Td8uQsXi9p0HWzDGsRmI7Dg3x-FBo3BWtzN5k-Pe8/edit?pli=1

Healthcare is far from realizing the Quintuple Aim of optimizing health at reduced costs while increasing equity and patient and clinician satisfaction. Aspiring Learning Health Systems (LHSs) are attempting to close this gap by enhancing the cycle whereby healthcare evidence is used to guide care—and care delivery results are used to continually improve available evidence. Interoperable, computable biomedical knowledge (CBK) is essential to optimize this cycle.

From 2018 to 2021, the AHRQ Evidence‐based care Transformation Support initiative (ACTS) engaged over 330 individuals from diverse stakeholder groups to produce a stakeholder‐driven future vision and action plan—and conduct pilots and concept demonstrations—for a knowledge ecosystem that supports CBK‐enabled LHS cycles. After ACTS, the project lead (Osheroff) formed the LHS Collaborative to drive stakeholder‐supported efforts to execute the ACTS action plan. The LHS Collaborative brings together diverse LHS stakeholders to enhance care transformation toward the Quintuple Aim and LHS function for clinical targets where improvements are urgently needed.

The Pain Management/Opioid Use LHS Learning Community (POLLC) is the first and most advanced LHS Collaborative community formed in follow‐up to the ACTS initiative. POLLC includes multiple stakeholder groups (e.g., care team members, guideline developers, health IT suppliers, quality improvement organizations, standards experts, and patient advisors) who are working together to identify and close pain and opioid management care gaps in care delivery organizations (CDOs). Other targets being addressed by public and private CDOs supported by LHS Collaborative tools and approaches include hypertension control, sickle cell disease management, venous thromboembolism (VTE) prophylaxis, and chronic kidney disease.

This Lightning Talk will illustrate how CBK‐related activities around the LHS cycle by the POLLC collaborative community and others could be integrated and enhanced to accelerate care transformation and LHS cycle function for this target. It will also outline how this approach could be scaled to address other high‐priority conditions by ensuring that various target‐focused LHS efforts contribute synergistically to building out a robust national/global knowledge ecosystem. In particular, by fostering a public‐private partnership to drive progress toward the shared future vision, as called for in the ACTS action plan.

The authors plan to leverage this lightning talk and related explorations to transition the potential opportunities to leverage CBK to improve LHS cycle efficiency and effectiveness into real world advancements. And do this in a manner that cultivates synergies with other CBK‐related activities to accelerate progress toward shared LHS goals.”

## NICE COMPUTABLE IMPLEMENTATION GUIDANCE—NEXT STEPS

Philip Scott, University of Wales Trinity Saint David

philip.scott@uwtsd.ac.uk

Charlie McCay, Ramsey Systems

charlie@ramseysystems.co.uk

Shaun Rowark, NICE

shaun.rowark@nice.org.uk

Background: The National Institute for Health and Care Excellent (NICE), a non‐departmental public body under the Department of Health and Social Care in England, has made a strategic commitment to producing clinical practice recommendations in a more modular and digital approach. In 2022–23, a first phase of work explored methods to convert narrative clinical recommendations to computable knowledge. The project involved NICE, MCBK‐UK and a broad stakeholder group from industry, academia and healthcare providers. Two in‐person ‘collaborathons’ were held, with fortnightly workstream calls in between, focused on NICE Guideline 28 (NG28), Type 2 diabetes in adults, specifically section 1.7 on drug treatment. The project adopted the World Health Organization (WHO) Digital Adaptation Kit (DAK) as a technology‐agnostic method to model clinical practice recommendations. A NICE Computable Implementation Guide (NCIG) following the structure of the DAK was produced for NG28 section 1.7, including user scenarios, personae, processes and workflow, core data elements and decision‐support logic. The first phase of work concluded in March 2023, with a recognition that further work was needed to demonstrate real‐world utility, define indicators (outcomes such as prescribing compliance) and functional/non‐functional requirements (such as data quality and usability) and to refine the documentation structure. Methods: The defined scope of the second phase started with real‐world implementation. The project was loosely coupled with concurrent work on the diabetes information standard commissioned by NHS England from the Professional Record Standards Body (PRSB), an umbrella body formed from the medical Royal Colleges and professional bodies from across health and care. This enabled multi‐disciplinary participation and drew upon PRSB's prior experience in development and implementation of clinical information standards. The selected implementation method was to define document templates (electronic forms) for primary care consultations by general practitioners (GPs) based on the core data elements and decision logic defined in the NCIG. GP document templates are a well‐established and relatively straightforward way of standardizing records of care, as they enable pre‐population from existing records, prompt for new data collection and can trigger links to practice workflows. Templates will also be defined for use in secondary care, and the effective sharing of information between primary and secondary care in support of the processes selected from NG28 and documented in the NCIG. Results: At the time of writing, GP template production is underway. By the time of the conference, we will report implementation experience of using the template with practices as well as outlining plans for outcome analysis and functional/non‐functional requirements. We will also be able to report on initial work in how this is supporting NICE's wider portfolio of guidance products.

## EVALUATION OF COMPUTABLE PERFORMANCE METRICS FOR CATARACT SURGERY ENABLED BY ARTIFICIAL INTELLIGENCE

Simmi Sen, Stanford University

simmi_sen@icloud.com

Eric Yeh, Stanford University

hsuhangyeh@gmail.com

Sophia Wang, Stanford University

sywang@stanford.edu

**Background**: Cataracts are caused by clouding of the natural lens that occurs with aging, leading to vision loss. Skilled completion of cataract surgery can result in almost immediate restoration of vision, but lengthy surgical time or poor instrument control may lead to prolonged or permanent vision loss. Cataract surgical trainees operate under the supervision of a preceptor who provides real‐time feedback which is typically of a qualitative nature. Until now, there has been no way to track important characteristics of surgical performance related to tool usage or eye positioning. We previously developed deep learning methods to recognize the locations of key anatomical landmarks such as the pupil and its center, as well as surgical instruments and their tips from cataract surgical video, allowing for the calculation of AI‐derived metrics related to performance, such as the total path length and area covered of individual tools, the velocity of tools, the centration of the eye, among others. The purpose of this study was to investigate whether these new AI‐computed cataract surgery performance metrics correlate with surgeon seniority and skill as rated by human experts.

**Methods**: 28 resident and 29 attending routine cataract surgical videos were anonymously collected. For each video, 6 machine‐generated metrics were generated by deep learning models: total path length, max velocity, area, phacoemulsification centration, eye fixation, and zoom level change. The former 3 metrics were individually calculated for limbus, pupil, and surgical instruments and the others were obtained at video level. Human raters independently rated the videos by Objective Structured Assessment of Cataract Surgical Skill (OSACSS), which had 20 subitems on a 5‐point scale with larger indicating better performance. Statistical differences of machine‐ and human‐rated scores between attending surgeons and trainees were tested by t tests, and the correlations between the two were examined by Pearson correlation coefficients.

**Results**: Phacoemulsification probe and irrigation/aspiration probe had significantly lower total path lengths, max velocities, and area metrics in attending videos. Attending surgeons exhibited better phacoemulsification centration and eye fixation. Most metrics correlated with average human‐rated OSACSS scores, including tool‐specific metrics and metrics related to microscope control (fixation: −.0.349; zoom level change: −0.322). Machine‐generated metrics with corresponding OSACSS subitems also exhibited significant negative correlations (fixation: −0.65, phacoemulsification probe area metric: −0.67).

Conclusion: Automatically generated AI‐metrics can be used to differentiate between attending and trainee surgeries and correlate with the human evaluation on surgical performance. These metrics can be automatically generated in a fast and scalable way in the post‐surgical analysis, enabling surgical trainees to receive useful feedback in a timely manner during their training. In addition, the numerical values of these metrics can be logged and reviewed later to track the improvement in different facets of surgical skills. The model shows promise in building a fully automatic, objective surgical feedback system in ophthalmology training which will allow for the standardized and consistent analysis of surgical techniques.

## ICU MORTALITY PREDICTION BASED ON PATIENT SUBGROUPS IDENTIFIED USING MULTIVARIATE TIME‐SERIES CLUSTERING

Tongyue Shi, National Institute of Health Data Science, Peking University

tyshi@stu.pku.edu.cn

Wentie Liu, National Institute of Health Data Science, Peking University

2211210777@stu.pku.edu.cn

Shuai Jin, Department of Adult Care, School of Nursing, Capital Medical University

jinshuai@ccmu.edu.cn

Jianguo Hao, National Institute of Health Data Science, Peking University, Institute of Medical Technology, Health Science Center of Peking University

jianguo.hao@bjmu.edu.cn

Huiying Zhao, Peking University People's Hospital

zhaohuiying109@sina.com

Guilan Kong, National Institute of Health Data Science at Peking University, Advanced Institute of Information Technology, Peking University

guilan.kong@hsc.pku.edu.cn

Data‐driven predictive analysis can help physicians in intensive care unit (ICU) to identify patients with high‐risk mortality at an early stage, facilitate the provision of personalized interventions, and thus may lead to improved patient outcomes. Most existing studies employed traditional logistic regression or machine learning methods to develop ICU mortality prediction models, and the extreme lab test results during the first 8 h or 24 h of ICU admission were used as data inputs. In fact, there is a high degree of heterogeneity among ICU patients, and the trends of vital signs may be different among patients with different mortality risk. Is it possible to classify patients into different subgroups and then evaluate the ICU mortality risk accordingly?

Patient subgroup analysis aims to put patients with similar characteristics into one subgroup. It helps physicians gain a better understanding of disease patterns, facilitates the provision of personalized treatment plans, and eventually, it may help optimize critical care and improve patient outcomes.

The Medical Information Mart for Intensive Care (MIMIC‐IV) database was used as data source. Time series data of five vital signs, including temperature, heart rate, mean arterial pressure (MAP), respiratory rate, and blood oxygen saturation (SpO2) were extracted for analysis.

An ensemble clustering model was developed for patient subgroup identification based on the time series vital signs data. It mainly comprised multivariate time‐series feature extraction and clustering methods. Firstly, Time2Feat was employed as the method for time‐series feature extraction. Then, different clustering algorithms including K‐Means, K‐Shape, and Agglomerative Hierarchical Clustering (AHC) were used as candidate clustering methods for subgroup analysis. In clustering, different distance metrics, including Euclidean distance, Dynamic Time Warping (DTW) and Soft‐DTW distance were tried. Finally, based on the Davies‐Bouldin Index (DBI) and Calinski‐Harabaz Index (CHI), a most suitable clustering model was selected, and the optimal number of clusters (k) was determined using the Elbow Method.

Furthermore, based on the identified subgroups, an ICU mortality prediction model was to be developed. Patient personal information including age, gender, and medical history, together with the patient subgroup were used for model development. Ideally, if the patient grouping and mortality prediction models can be deployed in practice, when a patient has been in ICU for 8 hr, the patient would be assigned to a specific subgroup and the corresponding ICU mortality risk would be generated in time.

To conclude, an ensemble patient grouping model based on multivariate time‐series vital signs data in the first 8 hr of ICU was developed in this study, and three distinct subgroups were identified. The development of an ICU mortality risk prediction model is still ongoing. Next step, we plan to implement the patient grouping and mortality prediction models in practice and evaluate real clinical effects.

## USING MACHINE LEARNING AND NATURAL LANGUAGE PROCESSING TO STRUCTURE MEDICAL EVIDENCE AND GRADE ITS QUALITY

Simon Šuster, University of Melbourne

simon.suster@unimelb.edu.au

Timothy Baldwin, Mohamed bin Zayed University of Artificial Intelligence

timothy.baldwin@mbzuai.ac.ae

Karin Verspoor, RMIT University

karin.verspoor@rmit.edu.au

Evidence‐based medicine relies on expert analysis of scientific findings to address various clinical questions. In addition to locating, organizing, and representing this knowledge/evidence in structured form, a critical aspect is determining its quality. This includes identifying potential biases in methodologies, reporting, and publication of individual studies, as well as assessing the strength of larger bodies of evidence. These steps are crucial for synthesizing reliable evidence. However, their complexity and time‐consuming nature hinder the accessibility of current medical knowledge.

In this talk, we will demonstrate how online collections of systematic reviews and published primary studies can be converted into machine readable datasets that capture key attributes of the evidence. These can then also be used for training and evaluating models for automated evidence assessment.

Relying on machine learning and natural language processing, these models can facilitate critical appraisal of medical evidence by not only predicting overall quality but also providing finer‐grained justification for different quality criteria.

Furthermore, we will touch upon the topics of reliability through calibration of classifier confidence, selective classification (i.e., decreasing classifier's error rate by sacrificing coverage), and algorithmic fairness (i.e., disparity in different performance measures) in the developed models. Addressing these concerns is essential to ensure that the created quality classifiers are both accurate and unbiased. We believe that in practice, such systems are most likely to work well by working alongside rather than replacing medical experts, who usually construct systematic reviews manually. In effect, an automated system can be seen as an additional assessor against which the manual assessment can be compared and potentially revised. As automated approaches to quality assessment become increasingly accurate and reliable in the future, fewer human examiners may be needed, potentially leading to time and resource savings.

By integrating machine learning and natural language processing, we work toward the MCKB vision of structuring knowledge and ensuring that it reflects the best and most current evidence and science. This in turn can empower healthcare professionals to make more informed decisions for their patients.

## EVIDENCE HUB: A PLACE TO EXCHANGE KNOWLEDGE AND FORM COMMUNITIES

Guy Tsafnat, PhD FAIDH, Founder and Chief Scientific Officer, Evidentli Pty Ltd

guyt@evidentli.com

Kenny Hong, PhD, Head of Cloud Engineering. Evidentli Pty Ltd

kenny.hong@evidentli.com

Evidence Hub is a public, free website specifically designed to host open‐source Computable Biomedical Knowledge Objects (CBKO). The site provides specific features to allow users define types of objects, provide applications to handle different objects, and form communities of interest around the objects.

Communities are provided with objected‐centered open, moderated discussions with the aim of reaching consensus about implementation, correct use, and logic of the CBKO.

Evidence Hub has three public interfaces: a moderated discussion forum, a source code repository, and an application programming interface (API). All three interfaces are free for anyone to use. Uploading and editing source code, contributing to discussions, and using the API require free registration. Source code published on the site through the API must comply with a JSON format derived from the Knowledge Grid format: computable source code, a human‐readable description, and a technical description of the execution environment for the object. In addition to these elements, the Evidence Hub format also has a meta‐data section that includes identifiers, contributors, and version information that Evidence Hub needs to effectively manage permissions and versions of the object.

The source code repository has a dedicated “technical” page for each CBKO with code browsing, version history, a download source‐code button, an option to create a “branch” copy of the object, and links to other technical resources such as technical documentation. Contributions to the source code can be uploaded, but inclusion in the object is subject to approval by the object's owner.

Each CBKO also has a “non‐technical” page. The main features of this page are a human‐readable description of the object and a discussion forum. The human readable description of the object is included in the object structure and is displayed here. The discussion forum is multi‐threaded and moderated by the object's owner.

Both pages have several common elements. Users that were logged in through a registered application (see API description below), will also see a one‐click import button on pages of CBKOs corresponding to the application. Social features such as sharing, posting, and watching CBKO's are also found on both pages.

The API provides similar functionality to the technical interface as well as endpoints to define new object types, and to register an application's capability to edit and/or execute an object of a particular type. The option to use the application will only be available to users already logged in through the application.

Knowledge hosted on the Evidence Hub remains with its owner. CBKOs must have a GPL 2.0 or compatible license. Evidence Hub is free of advertising and user information is only ever used to improve the Evidence Hub itself.

## USING REDCap™ TO SUPPORT THE DEVELOPMENT OF A LEARNING HEALTHCARE SYSTEM FOR MULTIPLE SCLEROSIS PATIENT MANAGEMENT

Minerva Viguera Moreno, Universidad Nacional de Educacion a Distancia (U.N.E.D)

minerva.viguera@gmail.com

María Eugenia Marzo‐Sola, Hospital San Pedro, Spain

memarzo@riojasalud.es

Fernando Martin‐Sanchez, Hospital Universitario La Paz

fmartinsanchez@salud.madrid.org

Multiple Sclerosis is a neurodegenerative disease which shows different phenotypes making difficult for clinicians to make short‐term decisions related with treatment and prognosis. Diagnosis is usually retrospective. Learning Healthcare Systems (LHS) can support clinical practice as they are devised as constantly improving modules. LHS can identify insights which allow evidence‐based clinical decisions and more accurate prognosis.

We are developing a LHS with the aim of reducing uncertainty. We are using REDCap™ to collect patients' data, both from Clinical Reported Outcomes (CRO) and from Patients Reported Outcomes (PRO). Once analyzed, this data will serve as a foundation to our LHS. We conducted bibliographical research to select those CRO and PRO collected in clinical practice or identified as possible risk factors. We designed a data collection and management protocol based on using REDCap™ which ensures anonymity and allows longitudinal and semi‐automated capture of health data. We are following a cohort of 300 patients for 18 months. At the moment, we have included 198 patients and received 141 complete responses and 7 partial responses regarding PROs baseline (70%). We are collecting PROs every 3 months; until the date, 59 patients have reach end this first endpoint: 32 answered and 27 have been requested by mail (54%). This data will be used to develop a LHS, able to accurate prognosis as well as to automatically include new data and improve its algorithm.

Although our results are preliminary, we are finding promising response rates from patients in the first round. On the other hand, rates from the second round are low and could compromise the longitudinal information needed for the LHS development.

## PROMPTING LARGE LANGUAGE MODELS TO CONSTRUCT BOOLEAN SEARCH STRATEGIES FOR GOOGLE AND ADVANCED SEARCH DATABASES

Hannah Wood, NHS England

hannah.wood@hee.nhs.uk

Large Language Models (LLMs) excel at language‐based tasks and can save considerable time if used appropriately and with skill. In this session, I will demonstrate that with careful prompting, LLMs like Bard and GPT‐4 can generate robust and comprehensive Boolean search strategies for use in Google and advanced search databases.

With urgent time pressures in healthcare becoming more prominent than ever before, and an ever‐increasing body of healthcare knowledge, LLMs provide an opportunity to save time without necessarily compromising quality. With newly accessible technologies, new search skills are required to responsibly search for healthcare topics.

While LLM responses are generally regarded as untrustworthy due to concerns around hallucination risks, bias and misinformation, language models are ideally placed to generate efficient and extensive Boolean search strategies for use in search databases. However, to achieve robust search strategies, knowledge of prompting tools for appropriate responses is required.

There are certain elements to avoid or be cognisant of when prompting LLMs to generate or develop search strategies: avoiding the generation of inaccurate Medical Subject Headings and search operators incompatible with search databases and ensuring that the search strategy itself is factually accurate.

Language models excel at tasks involving language; they can generate various synonyms and expand upon Boolean search blocks when prompted. These search blocks can be effectively used in Google and other search databases, improving search results, and locating relevant literature. They can also be useful for developing existing strategies.

With over 6 months of extensive testing, I conclude that language models have a place in the growing toolset of healthcare Knowledge and Library specialists, and those who frequently search the healthcare literature. LLMs can generate quality search blocks when prompted by a skilled searcher with an understanding of healthcare topics and experience with using Boolean logic in search databases; ultimately saving time and improving the quality of the searches.

## WHICH COMPUTABLE BIOMEDICAL KNOWLEDGE OBJECTS WILL BE REGULATED AS A MEDICAL DEVICE?

Jeremy C Wyatt, University of Southampton

j.c.wyatt@soton.ac.uk

Philip Scott, Programme Director, Institute of Management and Health, University of Wales Trinity Saint David

philip.scott@uwtsd.ac.uk

Matthew South, Director and Chief Technology Officer, OpenClinical CIC and Senior Software Architect at University of Birmingham

matt.south@openclinical.net

Mark Thomas, Associate Professor of Cardiology, University of Birmingham

m.r.thomas@bham.ac.uk

Caroline Jones, Associate Professor, Hillary Rodham Clinton School of Law, Swansea University

caroline.jones@swansea.ac.uk

Our aim in this project (sponsored by the UK MCBK chapter and the British Computer Society) was to understand which knowledge objects in a computable biomedical knowledge library are likely to be subject to regulation as a medical device in the UK. To achieve this understanding, a briefing paper was circulated to a multi‐disciplinary group of 25 people including regulators, lawyers, software engineers, digital health academics, librarians, and others with insights into knowledge management and device regulation. A one‐day workshop was then convened to discuss some key questions relating to our aim. Following wide ranging discussion by participants and further assessment of relevant regulations, a discussion paper was drafted by lead authors and circulated to other authors for their comments.

This contribution will report on how UK medical device regulators are likely to treat the different kinds of knowledge objects that may be stored in computable biomedical knowledge libraries. While our focus is on the likely approach taken by UK regulators, our UK regulators have contributed to the work of the International Medical Device Regulators Forum so our analysis will also be relevant to the approaches taken by regulators elsewhere.

We outline the UK criteria for medical devices and the responsibility of device manufacturers, then examine the regulatory implications for knowledge objects that correspond with each of the four knowledge levels described by Boxwala in 2011. We also propose an additional knowledge level for tagged fragments of guidelines etc. that we call level 2b, which is positioned between level 2 and level 3.

Our main conclusion is that if a knowledge object is directly executable and is described or marketed as being intended for a medical purpose to provide decision support, it will be in scope of UK regulation as “software as a medical device”. Conversely, if the knowledge object is not directly executable (e.g., it consists of an algorithm, a ruleset, an order set, pseudocode or some other knowledge representation) or the developers make no claim that the object can be used directly for a medical purpose (e.g., it is presented as pluripotential, so could be used to support medical research or education), it is not likely to be subject to UK regulation.

We expect similar reasoning to be applied in other countries with similar regulatory principles.

